# Supplementary material for: Temporal pattern recognition in retinal ganglion cells is mediated by dynamical inhibitory synapses
Source: Nat Commun. 2024 Jul 20;15:6118. doi: 10.1038/s41467-024-50506-7 (PMC11271269; doi:10.1038/s41467-024-50506-7)
Supplement: Supplementary file 1 — Supplementary Information [file 41467_2024_50506_MOESM1_ESM.pdf]

Supplementary Figures

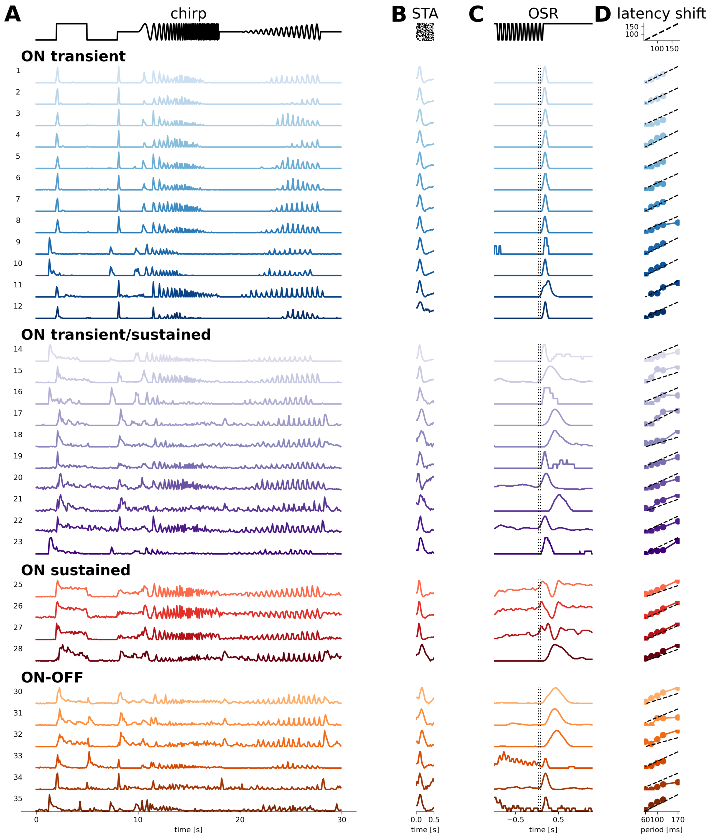

Figure S1: **ON cells that exhibit an OSR belong to various cell types.** Manual clustering of chirp responses from all ON cells with OSR yielded 4 broad cell types that show an OSR: ON transient, ON transient/sustained, ON sustained and ON-OFF. Shown are 35 cells with a response to the chirp stimulus and have a spike-triggered average to checherboard stimulation. 5 other cells which are included in the statistics in the main paper but do not have a chirp response or STA are not shown in this figure. **A.** Response traces to a chirp stimulus. **B.** Temporal spike triggered average obtained after STA analysis to white noise-checkerboard stimulus. **C.** OSR after control stimulation with 12 Hz flash train (firing rate). **D.** Scaling between response latency and stimulus period for frequencies of 6,8,10,12,16 Hz. Black line has a slope of 1 for reference. Source data are provided as a Source Data file.

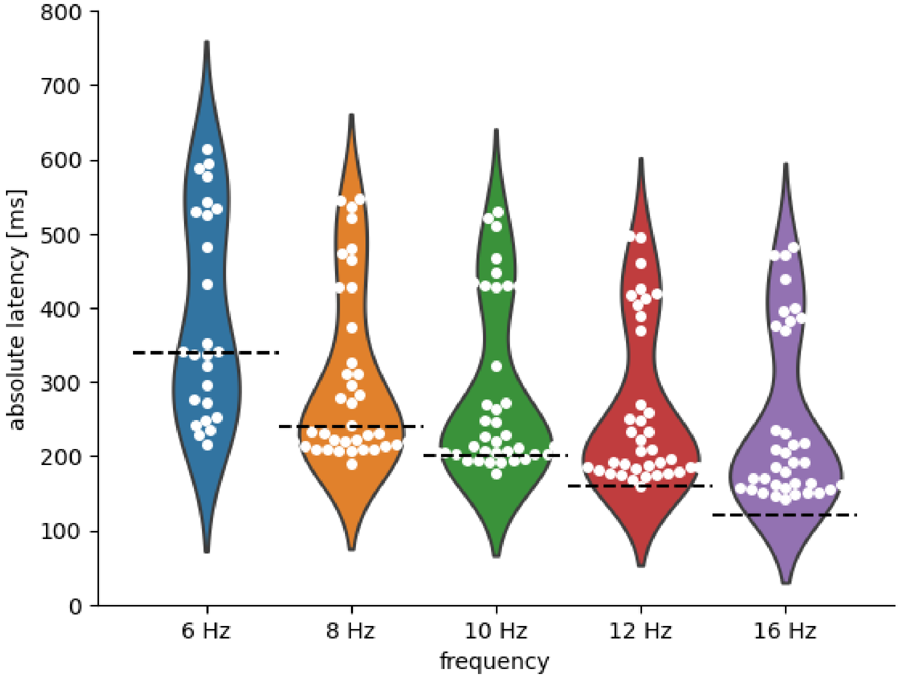

Figure S2: **Absolute latency of the OSR after all frequencie tested of n = 35 cells included in our study.** Shaded colored areas show the density, individual datapoints are shown in light blue. Dotted black lines indicate $2T$ for each period $T = \frac{1}{f}$. 6 Hz: max = 615 ms , min 216 ms, median = 340 ms; 8 Hz: max = 547 ms , min 189 ms, median = 232 ms; 10 Hz: max = 529 ms , min 178 ms, median = 220 ms; 12 Hz: max = 497 ms , min 159 ms, median = 207 ms; 16 Hz: max = 483 ms , min 142 ms, median = 188 ms; Source data are provided as a Source Data file.

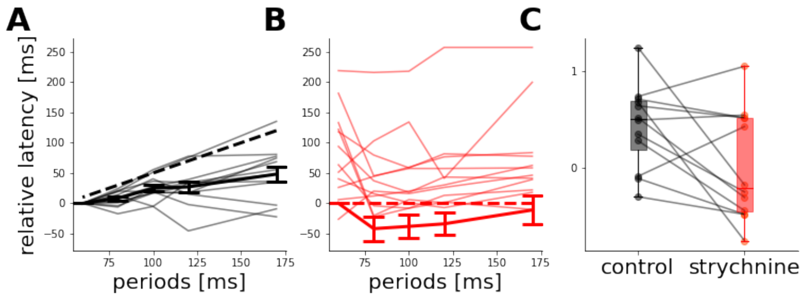

Figure S3: **Spiking onset is not a consistent quantity of the OSR latency shift.** **A.** Response onset latency against stimulus period in control conditions in all cells that show an OSR in their peak timing. Mean 0.39 ± 0.11, compared to thick dotted line which has a slope of 1. All latencies are referenced to the latency to the fastest frequency in control condition. **B.** Response onset latency against stimulus period in strychnine conditions in all cells that show a latency shift in their peak timing. Mean 0.15 ± 0.36, thick dotted line has a slope of 0. All latencies are referenced to the latency of the fastest frequency in control condition. **C.** Slope quantification, no significant difference slope in response onset between control and strychine, p = 0.056. Source data are provided as a Source Data file.

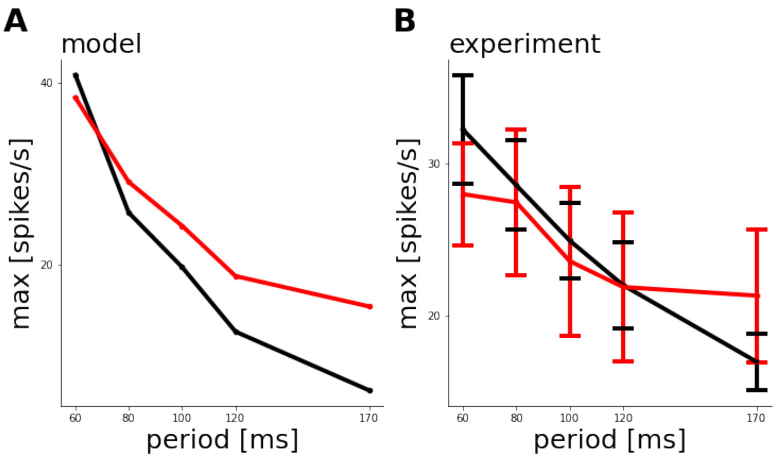

Figure S4: **Peak amplitude to different stimulus frequencies are not systematically impacted by strychnine. A.** Amplitude of the OSR against stimulus period for control and strychnine conditions in simulations. **B.** Same for experimentally measured amplitudes, not significantly different after Bonferroni-Holm correction **?** (6 Hz: p = 0.26, 16 Hz: p = 0.975).Source data are provided as a Source Data file.

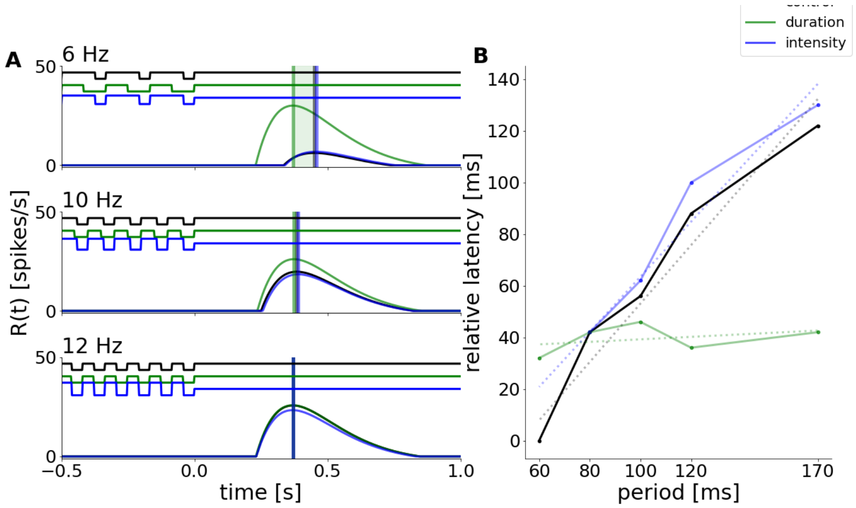

Figure S5: **Model predicts the selectivity of latency scaling to transient periodic flashes. A.** Simulated response traces of the model to flash trains of 3 different frequencies in control condition (black) and with modulations of flash-duration (green) and inter-flash brightness (blue). Stimuli are shown in the upper panels, simulated firing rate below. Vertical lines highlight the peak time-point, shaded areas show the latency shift compared to the control response. **B.** Latency of the OSR plotted against stimulus period for the 3 stimulus types. Solid lines are datapoints, dotted lines show linear fit between latency and period. Slope control: 1.13, slope duration modulation: 0.05, slope intensity modulation: 1.06.

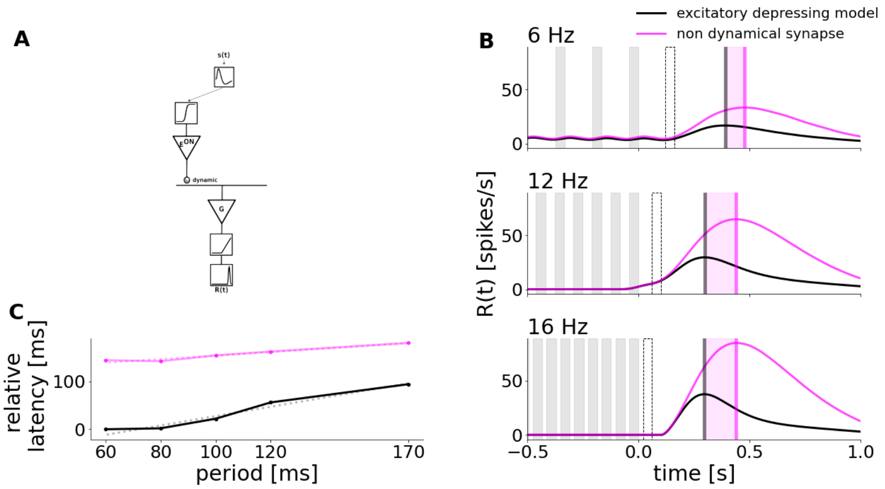

Figure S6: **Model with a biphasic ON bipolar cell can simulate the latency shift of the OSR via excitatory plasticity.** **A.** Schematic description of the model with one biphasic ON unit that has a plastic synapse. **B.** Response traces to 3 stimulus frequencies with the purely excitatory model with plasticity (black) compared to a model without plasticity (magenta). **C.** Scaling between response latency and stimulus period in both model variations. Slope full model: 0.98, slope without plasticity: 0.37. Parameter used for the simulation : $\tau_{\mathrm{OPL}} = 0.1$, $\tau_{\mathrm{OPL2}} = 0.1$, $\tau_{\mathrm{E^{ON}}}$, $\tau_{\mathrm{G}} = 0.05$, $a_{\mathrm{ON}} = 14.0$, $b_{\mathrm{ON}} = 0.0$ $w_{\mathrm{E^{ON}}} = 200.0$, $S_{\mathrm{E^{ON}}} = 0.5$, $\theta_{\mathrm{E^{ON}}} = 0$, $k_{\mathrm{rel}} = 50.3$, $k_{\mathrm{rec}} = 1.0$, $\beta = 13.6$, $\theta_{\mathrm{G}} = 0.0$, $s_{\mathrm{G}} = 2200$. Source data are provided as a Source Data file.

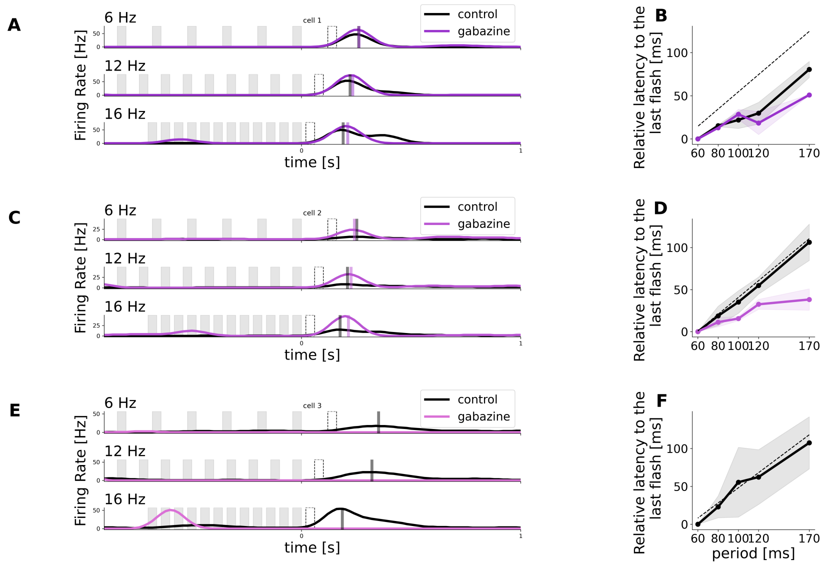

Figure S7: Gabazine has various effects on the OSR. **A.,C.,E.** Response traces to flash trains of 3 different frequencies in control (black) and gabazine (shades of purple) conditions. **B.,D.,E.** Mean relative latency to the last flash of the stimulus plotted against stimulus period for control (black) and gabazine (shades of purple) conditions. Dotted line shows slope of 1. **A.,B.** Example cell where OSR remains largely unaffected by gabazine. (Slope control = 0.71, gabazine = 0.4., n = 2 cells) **C.,D.** Example cell where OSR slope decreses with gabazine. (Slope control = 0.97, slope gabazine = 0.35, n = 4 cells). **E.,F.** Example cell where OSR dissapears though gabazine. (Slope control = 0.96, n = 4 cells). Source data are provided as a Source Data file.
